# Relationship between Subclinical Thyroid Dysfunction and the Risk of Cardiovascular Outcomes: A Systematic Review and Meta-Analysis of Prospective Cohort Studies

**DOI:** 10.1155/2017/8130796

**Published:** 2017-08-31

**Authors:** Jing Sun, Liang Yao, Yuan Fang, Ruifei Yang, Yaolong Chen, Kehu Yang, Limin Tian

**Affiliations:** ^1^Department of Endocrinology, Gansu Provincial Hospital, Dong Gang West Road, Lanzhou, Gansu 730000, China; ^2^Clinical Evidence-Based Medicine Center, Gansu Provincial Hospital, Dong Gang West Road, Lanzhou, Gansu 730000, China; ^3^Evidence-Based Medicine Center, School of Basic Medical Sciences, Lanzhou University, Lanzhou 730000, China

## Abstract

**Background:**

Evidence on the association between subclinical thyroid dysfunction and the risk of cardiovascular outcomes are conflicting.

**Methods and Results:**

PubMed, EMbase, Web of Science, Cochrane Library, and China Biology Medicine (CBM) databases were searched from inception to July 10, 2016. A total of 16 studies were included for meta-analysis. We found that subclinical hypothyroidism was not correlated with coronary heart disease (CHD) (RR = 1.17; 95% CI, 0.91–1.52), total mortality (RR = 1.02; 95% CI, 0.93–1.13), cardiovascular mortality (RR = 1.06; 95% CI, 0.77–1.45), heart failure (RR = 1.17; 95% CI, 0.87–1.57), and atrial fibrillation (RR = 1.05; 95% CI, 0.91–1.21), except CHD mortality (RR = 1.37; 95% CI, 1.03–1.84). Subgroup analysis indicated a higher estimation risk in CHD (RR = 1.54; 95% CI, 1.00–2.39), cardiovascular mortality (RR = 2.14; 95% CI, 1.43–3.22), and CHD mortality (RR = 1.54; 95% CI, 1.11–2.15) among participants < 65 years. Furthermore, subclinical hyperthyroidism was found to be associated with CHD (RR = 1.20; 95% CI, 1.02–1.42), total mortality (RR = 1.27; 95% CI, 1.07–1.51), and CHD mortality (RR = 1.45; 95% CI, 1.12–1.86).

**Conclusions:**

Subclinical hypothyroidism is likely associated with an increased risk of CHD mortality, and subclinical hyperthyroidism is likely associated with increased risk of CHD, CHD mortality, and total mortality.

## 1. Introduction

Subclinical thyroid dysfunction (SCTD) is defined as an abnormal serum thyroid-stimulating hormone (TSH) level, with normal concentrations of free thyroxine (T4) and free triiodothyronine (T3) [1, 2]. The prevalence of SCTD was high in the general population: 3.1 ~ 8.5% among patients with subclinical hypothyroidism [3] and 1 ~ 10% among those with subclinical hyperthyroidism [4–7]. Subclinical hypothyroidism is more common than subclinical hyperthyroidism [8]. However, early screening and treatment of SCTD are still controversial on the basis of current evidence [1, 8].

The cardiovascular system is one of the main target organs of thyroid hormones [9], and elevated or decreased TSH levels can adversely affect the cardiovascular system [10, 11]. Some studies demonstrated that subclinical hypothyroidism increased cardiovascular risk factors including altered lipid profile [12], insulin resistance [13], oxidative stress [14], increased vascular stiffness [15], and endothelial dysfunction [16]. Subclinical hyperthyroidism also manifested in the cardiovascular system by numerous ways, including increased heart rate [17], left ventricular mass [18], susceptibility to endothelial dysfunction [19], and left ventricular diastolic dysfunction [20], which can cause changes in cardiovascular morphology and function.

However, there is conflicting evidence regarding the association between coronary heart disease (CHD) and mortality [5, 21–24]. Although several meta-analyses had been published, they included studies prior to 2011 and revealed conflicting findings [25–30]. In recent years, several prospective cohort studies with a large sample size have been published in peer-reviewed journals [21, 31–35], which may change findings of previous meta-analyses. So it is necessary to conduct a new meta-analysis to explore the relationship between SCTD and the risk of cardiovascular outcomes to provide more rigorous and credible evidence for the clinical decision related to SCTD.

## 2. Material and Methods

### 2.1. Search Strategy

We systematically searched PubMed, EMbase, Web of Science, Cochrane Library, and China Biology Medicine (CBM) databases for target studies from inception to July 10, 2016, without language restriction. The search strategy was conducted by medical subject headings (thyroid diseases, hypothyroidism, hyperthyroidism, thyrotropin, thyroid hormones, myocardial ischemia, mortality, survival, heart failure, atrial fibrillation, and cardiovascular diseases) and with text words (subclinical hypothyroidism, subclinical hyperthyroidism, subclinical dysthyroidism, and subclinical thyroid). In addition, we also searched the references of included articles to expand the search.

### 2.2. Study Selection

Two reviewers (Jing Sun and Ruifei Yang) independently screened the abstracts and titles of the searched articles according to the inclusion criteria: (1) reported diagnostic criteria for SCTD; (2) must be full-text prospective cohort study; (3) at least reported one cardiovascular outcome; (4) and compared with euthyroidism. The following articles were excluded: (1) studies including only overt thyroid dysfunction or lacking a report on SCTD diagnostic criteria; (2) case series or reviews and conference abstracts; (3) raw data could not be extracted; (4) and similar studies were reported without additional data to analyze and extract.

### 2.3. Data Extraction

Two reviewers (Jing Sun and Ruifei Yang) independently extracted data on participant characteristics from the selected studies in a standardized data extraction form. We extracted the following information from each included article: the name of the first author, year of publication, number of participants, mean follow-up duration, participant characteristics, baseline of thyroid function and cardiovascular risk factors (e.g., smoking, diabetes, blood pressure, and low- and high-density lipoprotein cholesterol level), (thyroid) medication use at baseline, and outcome data.

### 2.4. Methodological Quality Assessment of Included Studies

Methodological quality assessment was performed in accordance with the Newcastle-Ottawa Quality Assessment Scale (NOS), which is a validated scale for nonrandomized studies in meta-analyses [36]. In this analysis, studies that received ≥7 points were considered of high methodological quality. Besides, we assessed some other items to evaluate the methodological quality of included studies: (1) whether the study was population based; (2) whether the study had formal adjudication procedures, which were defined as having clear outcome criteria that were reviewed by experts for each potential case; (3) whether the adjudication was performed without knowledge of thyroid status; and (4) the adjustments for multivariate analysis.

### 2.5. Quality of the Evidence Assessment

We assessed the quality of evidence for each outcome using the Grading of Recommendations Assessment, Development and Evaluation (GRADE) criteria [37]. We rated the quality of evidence, starting at high quality and downgrading for the risk of bias, imprecision, inconsistency, indirectness, and publication bias. In this meta-analysis, we present “Summary of findings” table using the following primary outcomes: (1) CHD, (2) total mortality, (3) cardiovascular mortality, (4) CHD mortality, (5) heart failure, and (6) atrial fibrillation.

### 2.6. Data Synthesis and Statistical Analysis

In this meta-analysis, risk ratio (RR) and 95% confidence interval (CI) were considered as the effect size for all studies. Forest plots were produced to visually assess the RR and corresponding 95% CI using random-effects models. In addition, we also conducted a fixed-effects model for comparison. Heterogeneity between studies was assessed via the forest plot, while *I*^2^ values described the total variation between studies. *I*^2^ values of <25%, 25%–50%, and >50% indicated low, moderate, and high heterogeneity, respectively. We used sensitivity and subgroup analyses to explore and interpret the sources of high heterogeneity. We explored the relationship of SCTD and cardiovascular outcomes among participants of different age groups (<65 years or ≥65 years and <60 years, 60–79.9 years, or ≥80 years) and different TSH levels (4.5–9.9 mIU/L or 10–19.9 mIU/L). Statistical analysis was performed with STATA software, version 12.0 (StataCorp, College Station, TX).

## 3. Results

### 3.1. Study Selection

**Figure 1:**
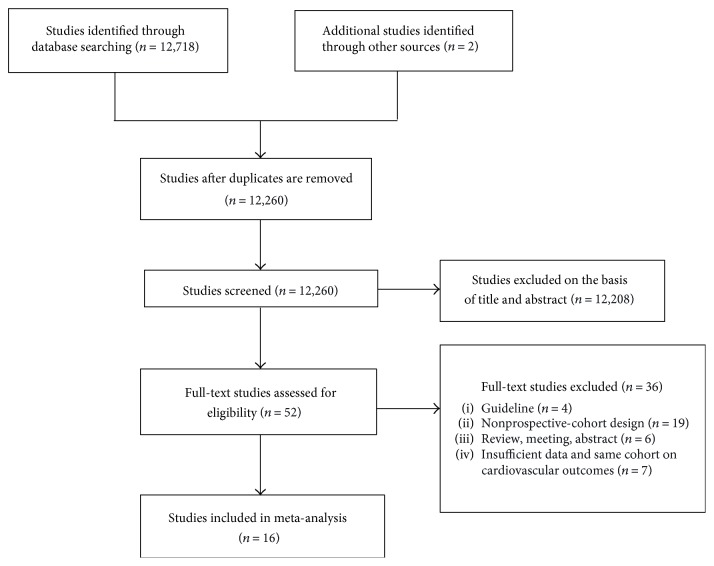
Flow chart for study inclusion.

A total of 12,720 studies initially identified, and 12,260 remained after excluding duplicate citations. 12,208 studies were excluded by title and/or abstract not related to the research topic, and 36 studies were excluded by full-text screening. Finally, 16 studies that met the eligibility criteria were included for further analysis. The literature screening process is shown in Figure 1.

### 3.2. Characteristics and Quality of Included Studies

Table 1 shows the characteristics of included studies in our meta-analysis. A total of 71,808 participants were included in the 16 studies, of which 5178 were defined as having subclinical hypothyroidism (7.2%), 64,330 with euthyroidism (89.6%), and 2300 with subclinical hyperthyroidism (3.2%). 15 studies reported an association between subclinical hypothyroidism and cardiovascular outcomes [5, 21, 23, 24, 31–35, 38–43], and 13 reported an association between subclinical hyperthyroidism and cardiovascular outcomes [5, 21, 23,24, 32–35, 39, 40, 42–44]. Most of the participants were middle aged or elderly (aged 45.4–85 years), and the follow-up duration of the included studies ranged from 3.2 to 20 years. All studies were population based, and seven studies reported adjudication without knowledge of thyroid status (Supplemental Material Table 1 available online at https://doi.org/10.1155/2017/8130796). Based on the NOS results, the studies showed similar scores overall but scored differently on the separate item (Supplemental Material Table 2). The TSH cutoff values were controversial in different studies, ranging from 4.0–6.0 mIU/L in subclinical hypothyroidism and 0.3–0.5 mIU/L in subclinical hyperthyroidism.

### 3.3. Main Results

#### 3.3.1. Subclinical Thyroid Dysfunction and CHD

**Figure 2:**
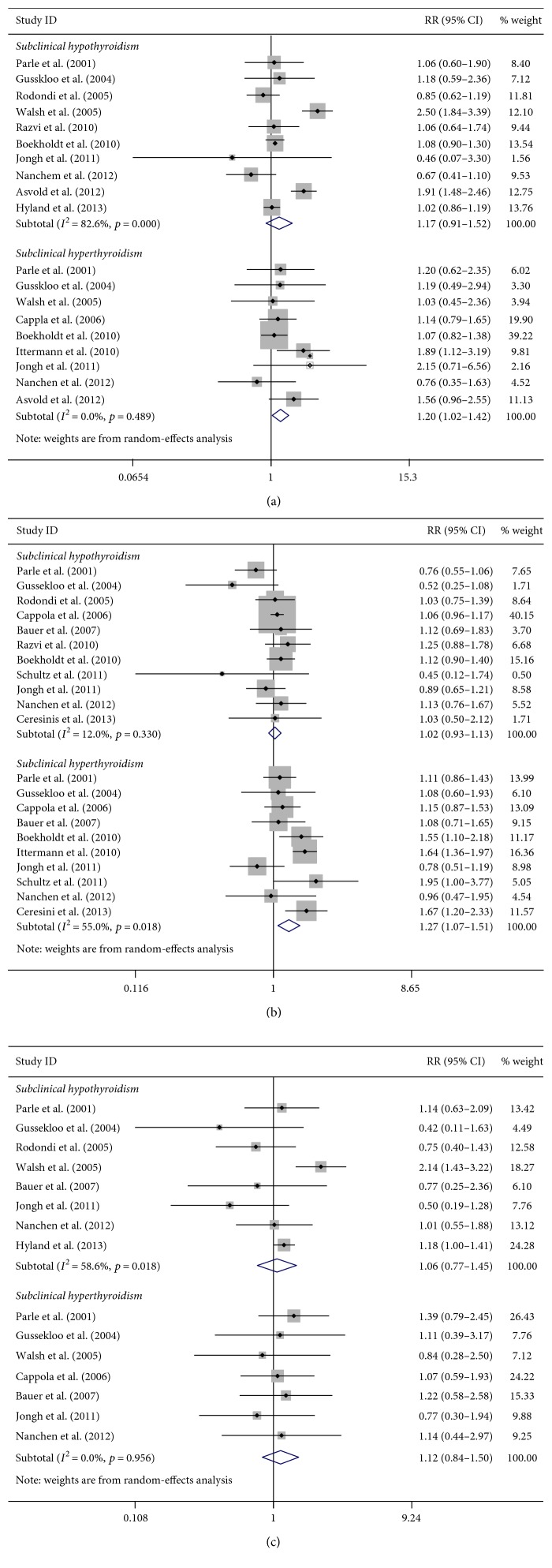
Forest plots for subclinical thyroid dysfunction. (a) Forest plots for subclinical thyroid dysfunction and risk of CHD; (b) forest plots for subclinical thyroid dysfunction and risk of total mortality; (c) forest plots for subclinical thyroid dysfunction and risk of cardiovascular mortality.

Ten studies [5, 21, 24, 31, 33, 34, 38, 41–43] (4979 CHD events) reported a relationship between subclinical hypothyroidism and CHD (Figure 2(a)). The pooled RR was 1.17 (95% CI, 0.91–1.52), and the heterogeneity was high (*p* < 0.01, *I*^2^ = 82.6%). Risk was similar after adjusting cardiovascular risk factors (Table 2). Heterogeneity was reduced by excluding studies that included thyroid hormone recipients (*p* = 0.68, *I*^2^ = 0.0%).

Nine studies [5, 21, 23, 24, 33, 34, 42–44] (4116 CHD events) demonstrated the relationship between subclinical hyperthyroidism and CHD (Figure 2(a)). The result of meta-analysis showed a significant association (RR = 1.20; 95% CI, 1.02–1.42), and no heterogeneity was found (*p* = 0.49, *I*^2^ = 0%). However, no significant result was found after adjusting cardiovascular risk factors and excluding studies with antithyroid drug recipients (Table 3).

#### 3.3.2. Subclinical Thyroid Dysfunction and Total Mortality

No significant association was found in total mortality and subclinical hypothyroidism (RR = 1.02; 95% CI, 0.93–1.13), and the result was pooled from eleven studies [5, 21, 23, 32, 33, 35, 38, 40–43] (Figure 2(b)). Sensitivity analyses yielded similar results after excluding studies without adjusting analysis for cardiovascular risk factors, studies with a particular population (mean age ≥ 80 years), and studies containing thyroid hormone recipients (Table 2).

For subclinical hyperthyroidism, the pooled result showed an increased total mortality (RR = 1.27; 95% CI, 1.07–1.51) based on ten studies [5, 21, 23, 32, 33, 35, 40, 42–44] (Figure 2(b)). Risk was similar after excluding thyroid hormone recipients (Table 3).

#### 3.3.3. Subclinical Thyroid Dysfunction and Cardiovascular Mortality

Eight studies [21, 24, 31, 33, 40–43] (1755 cardiovascular mortality events) reported a relationship between subclinical hypothyroidism and cardiovascular mortality (Figure 2(c)). We found that subclinical hypothyroidism was not significantly associated with cardiovascular mortality (RR = 1.06; 95% CI, 0.77–1.45). Risk was similar after adjusting cardiovascular risk factors (Table 2).

Seven studies [21, 23, 24, 33, 40, 42, 43] (1347 cardiovascular mortality events) reported the relationship between subclinical hyperthyroidism and cardiovascular mortality (Figure 2(c)). The pooled RR for cardiovascular mortality was 1.12 (95% CI, 0.84–1.50), without heterogeneity (*p* = 0.10, *I*^2^ = 0.0%). Most sensitivity analyses yielded similar results (Table 3).

#### 3.3.4. Subclinical Thyroid Dysfunction and CHD Mortality

**Figure 3:**
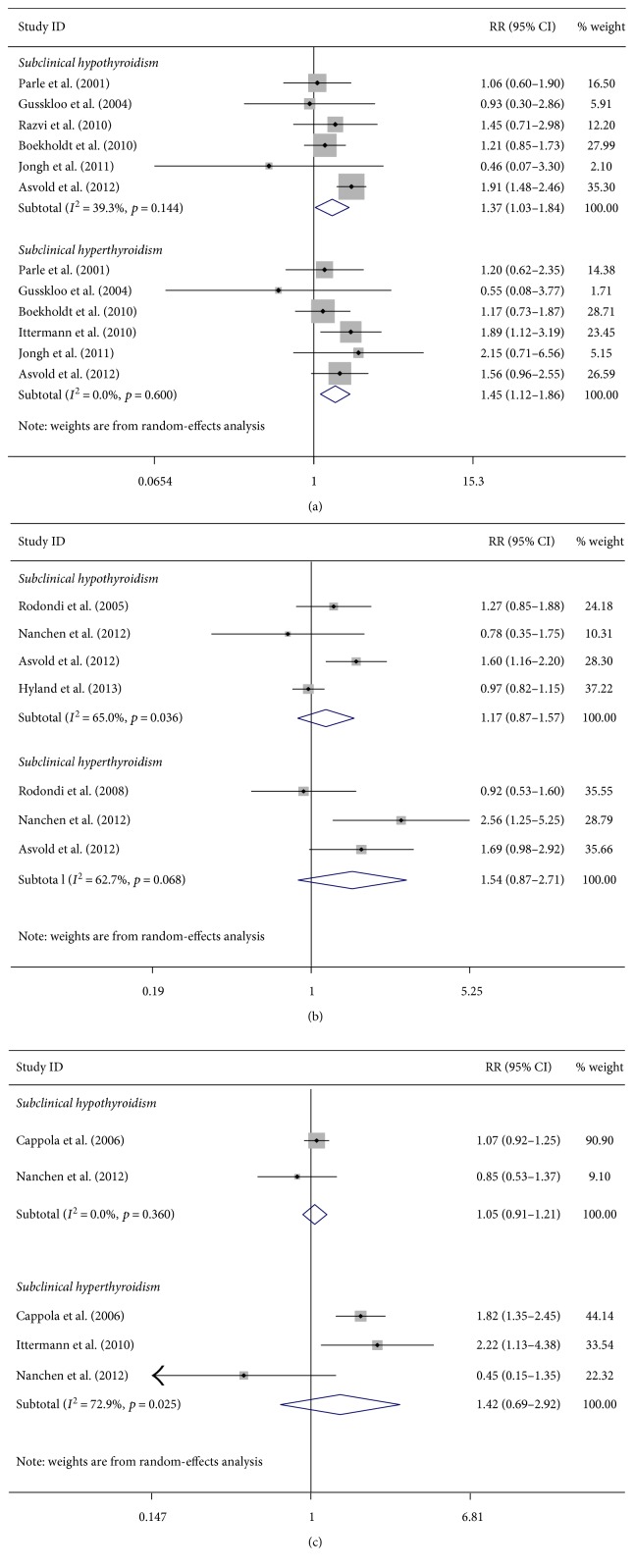
Forest plots for subclinical thyroid dysfunction. (a) Forest plots for subclinical thyroid dysfunction and CHD mortality; (b) forest plots for subclinical thyroid dysfunction and risk of heart failure; (c) forest plots for subclinical thyroid dysfunction and risk of atrial fibrillation.

Compared with euthyroidism, subclinical hypothyroidism was associated with CHD mortality (RR = 1.37; 95% CI, 1.03–1.84) based on six studies [5, 21, 34, 38, 42, 43] (1369 CHD mortality events) (Figure 3(a)). No statistical association was found between subclinical hypothyroidism and CHD mortality after adjusting cardiovascular risk factors and excluding studies included antithyroid drug recipients (Table 4).

We found that subclinical hyperthyroidism was associated with CHD mortality (RR = 1.45; 95% CI, 1.12–1.86) based on six studies [5, 21, 34, 42–44] (1263 CHD mortality events) (Figure 3(a)). Sensitivity analyses yielded similar results after excluding studies without further adjustment analysis for cardiovascular risk factors, studies with a particular population (mean age ≥ 80 years), and studies including antithyroid drug recipients (Table 4).

#### 3.3.5. Subclinical Thyroid Dysfunction, Heart Failure, and Atrial Fibrillation

Among participants with subclinical hypothyroidism, there happened 1704 heart failure events with pooled RR of 1.17 (95% CI, 0.87–1.57) and 1339 atrial fibrillation events with pooled RR of 1.05 (95% CI, 0.91–1.21) (Figures 3(b) and 3(c)).

For subclinical hyperthyroidism, there happened 1200 heart failure events with pooled RR of 1.54 (95% CI, 0.87–2.71) and 1240 atrial fibrillation events with pooled RR of 1.42 (95% CI 0.69–2.92) (Figures 3(b) and 3(c)).

#### 3.3.6. Subgroup Analysis

Subgroup analyses about the association between subclinical thyroid dysfunction and CHD, CHD mortality, cardiovascular mortality, and total mortality in different age and TSH levels were shown in Tables 2–4.

## 4. Discussion

This meta-analysis was a systematic literature review for prospective cohort studies to ensure the relationship of SCTD and cardiovascular outcomes. We included 16 prospective cohort studies, and we found that subclinical hypothyroidism was likely associated with increased risk of CHD mortality and subclinical hyperthyroidism was likely associated with increased risk of CHD, CHD mortality, and total mortality. Although the discovery of SCTD might increase the risk of other cardiovascular outcomes, the difference was not significant. For subclinical hypothyroidism, the quality of evidence was moderate for total and CHD mortality, low for CHD, cardiovascular mortality, heart failure, and atrial fibrillation (Supplemental Material Table 3). For subclinical hyperthyroidism, the quality of evidence was moderate for CHD, CHD mortality, total, and cardiovascular mortality and low for heart failure and atrial fibrillation (Supplemental Material Table 4).

For the association between subclinical hypothyroidism and CHD, total, and cardiovascular mortality, our results are consistent with previous meta-analyses [27, 28]. Although some meta-analyses [29, 45] reported significant associations between subclinical hypothyroidism and CHD, previous meta-analyses included case-control and cross-section studies, which could not accurately reflect a cause-and-effect relationship. Recent meta-analysis showed that the association between CHD and subclinical hypothyroidism was TSH related, and this phenomenon was significantly elevated in individuals with TSH levels of 10 mIU/L or greater [27]. However, this was not confirmed by our analysis. For TSH values over 10 mIU/L, our meta-analysis showed no significant association (RR = 1.38; 95% CI, 0.68–2.78) between subclinical hypothyroidism and CHD based on 12,731 participants. However, Rodondi et al. [27] found a positive results in CHD (HR = 1.89; 95% CI, 1.28–2.80) based on 235 participants, which was at odds with our meta-analysis. Our result was based on perspective cohort studies with NOS score > 7, so we believed that our result was more reliable because of low risk of bias, large sample size, and statistical power. For TSH values below 10 mIU/L, our meta-analysis got a similar result with previous meta-analyses [27, 28]. In overall pooled data, we found high heterogeneity for CHD. To detect the issue of heterogeneity, we performed subgroup and sensitivity analyses. Heterogeneity was reduced by excluding studies that included thyroid hormone recipients.

Our study found that in patients with mean age < 65 years, subclinical hypothyroidism significantly increased the risk of CHD, CHD mortality, and cardiovascular mortality. However, these results were not obtained in patients aged >80 years on average. Owing to the relatively few studies, the results should be interpreted carefully and confirmed by further studies. We found that subclinical hypothyroidism was associated with a slightly, but not significantly, increased risk of atrial fibrillation and heart failure. However, the results should be considered carefully owing to the small sample size.

For the associations between subclinical hyperthyroidism and cardiovascular outcomes, our study demonstrated that the risk of CHD, CHD mortality, and total mortality was increased in patients with subclinical hyperthyroidism, and the difference was significant. Three possible mechanisms have been reported to explain this association. Firstly, insulin resistance is the major risk factors for cardiovascular disease [46]. Insulin sensitivity was significantly decreased in patients with subclinical hyperthyroidism, thereby increasing the risk of cardiovascular events [47–49]. Secondly, the levels of plasma fibrinogen and D-dimer in subjects with subclinical hyperthyroidism were higher than those with euthyroidism [50]. Elevated fibrinogen is an independent risk factor for coronary artery disease, and elevated serum D-dimer may increase the risk of future myocardial infarction. Lastly, several cardiovascular mechanisms have been reported in subclinical hyperthyroidism, such as increasing heart rate [17], left ventricular mass [18], being prone to endothelial dysfunction [19], and left ventricular diastolic dysfunction [20], causing cardiovascular changes in morphology and function. One study has reported that subclinical hyperthyroidism participants with decreased serum TSH levels had higher intima media thickness (IMT) than those with euthyroidism [51].

Our study found an increased CHD mortality (three studies) and total mortality (two studies) in patients with subclinical hyperthyroidism < 65 years. However, this potential difference should be interpreted with caution, because there were only few studies reported these outcomes. Selective bias might be existing in the other studies, which could contribute to a misleading result. On the other hand, the duration of follow-up could also influence the result. An increased total mortality was observed in a study of people > 60 years with subclinical hyperthyroidism, during the first five years of follow-up [43]. Another prospective observational study from Brazil found that all-cause and cardiovascular mortality were also significantly higher in individuals with subclinical hyperthyroidism, during a 7.5-year follow-up [22]. However, in these two studies, the increased mortality emerged during the <10-year follow-up, whereas it was not observed over the full follow-up period of 10 years [22, 43]. In our study, the follow-up duration ranged from 8.5 to 12.3 years in CHD mortality and 8.5 to 10.6 years in total mortality.

In the age subgroup analysis, in those mean age < 65 years, the risk of CHD, CHD mortality, cardiovascular mortality, and total mortality is higher than for those of average age ≥ 65 years. This showed that age is a risk factor for cardiovascular outcomes, suggesting that subclinical thyroid dysfunction in patients with mean age < 65 years should pay attention to primary and secondary prevention of cardiovascular disease. The risk of cardiovascular outcomes in patients of average age ≥ 80 years was relatively low, possibly owing to other diseases such as cancer, mortality, or other diseases.

Subclinical thyroid dysfunction is a common endocrine and metabolic disease, characterized by abnormal TSH level. Subclinical thyroid dysfunction has become more easily to be found in the population along with the continuous improvement of detection. However, the clinical symptoms of subclinical thyroid dysfunction are slight and uneven and can easily go unnoticed which might lead to controversy on screening and treatment of subclinical thyroid dysfunction in practice [8, 52]. Our study found that subclinical thyroid dysfunction may increase cardiovascular risk; however, unlike dyslipidemia, diabetes mellitus, and hypertension, it may have a greater impact on cardiovascular risk. Clinical studies have shown that treatment of subclinical hypothyroidism is beneficial to endothelial function [16] and carotid arterial IMT [53], as these are early signs of atherosclerosis. Levothyroxine is commonly recommended as appropriate when the TSH concentration is ≥10 mIU/L. The recent available guidelines for the management of patients with serum TSH < 10 mIU/L should consider the age of the patient, associated risk factors, and comorbid conditions [54]. Treatment could be considered in patients aged >65 years with TSH levels from 0.1 to 0.39 mIU/L and might also be reasonable in symptomatic patients aged <65 years with TSH levels < 0.1 mIU/L [55].

Our study has several strengths. Firstly, we conducted a systematic literature search for potential studies, without language restrictions, as broadly as possible to include all studies related to the research topic. Secondly, we included only prospective cohort studies with NOS score ≥ 7 points, which could ensure the quality of our study. Besides, we assessed the quality of evidence using GRADE approach, which could reflect our confidence in the credibility of the results. Thirdly, we conducted subgroup and sensitivity analyses to various outcome measures.

Our analysis also has some limitations. Firstly, all data from this study were derived from observational studies, so the study may be affected by confounding factors and the results should be interpreted with caution. Secondly, thyroid function testing was measured at baseline, and we have no data on the possible progression from subclinical to overt dysfunction. In addition, different TSH cutoff levels in the original study, definitions of CHD, and diagnostic criteria are not uniform, and the adjustment of the confounding factors were different.

## 5. Conclusion

In summary, our meta-analysis showed that subclinical hypothyroidism is likely associated with an increased risk of CHD mortality, and subclinical hyperthyroidism is likely associated with increased risk of CHD, CHD mortality, and total mortality. Although there are some confounders in this study, for example, the associations found in most cases are much weak, varied follow-up duration and selection bias were suspected in most studies, which may weaken the results of this meta-analysis, we believe that the findings in this meta-analysis provide useful information for stakeholders with an interest in the outcomes of patients with subclinical thyroid dysfunction.

## Supplementary Material

Supplemental Material Table 1. Quality Assessment of Included Studies. Supplemental Material Table 2. Newcastle Ottawa Quality Assessment Scale for Cohorts of included studies. Supplemental Material Table 3.SUMMARY OF FINDINGS FOR THE MAIN COMPARISON. Supplemental Material Table 4: SUMMARY OF FINDINGS FOR THE MAIN COMPARISON.

## Figures and Tables

**Table 1:** Baseline characteristics of subclinical thyroid dysfunction in included studies.

First author, year	Sample	Mean age (range) (y)	Follow-up	TSH cutoff value (mIU/L) (Shypo/Shyper)	Thyroxine measured (Shypo/Shyper)	Exclusion of thyroid hormone/antithyroid drug recipients	Outcome (Shypo or Shyper)
Parle et al. [43]	1187	70.4 (>60)	8.2 y	>5.0/<0.44	Yes/yes	Yes/yes	CHD, CHD mortality, total, and cardiovascular mortality
Gussekloo et al. [42]	527	85	3.7 y	>4.8/<0.3	Yes/yes	Yes/yes	CHD, CHD mortality, total, and cardiovascular mortality
Rodondi et al. [41]	2730	74.7 (70–79)	4 y	≥4.5/NA	Yes/NA	NR/NA	CHD, HF, total, and cardiovascular mortality
Walsh et al. [24]	1890	49.8 (17–89)	20 y	>4.0/<0.4	Yes/yes	NR/NR	CHD and cardiovascular mortality
Cappola et al. [23]	3182	72.7 (≥65)	12.5 y	>4.5/<0.45	Yes/yes	Yes/yes	CHD, AF, total, and cardiovascular mortality
Bauer et al. [40]	487	71.7 (≥65)	11.9 y	>5.5/≤0.5	NR/NR	NR/NR	Total and cardiovascular mortality
Rodondi et al. [39]	3044	72.6 (≥65)	15 y	>4.5/<0.45	Yes/yes	Yes/yes	HF
Razvi et al. [38]	2350	45.4 (18–92)	20 y	>6/NA	NR/NA	Yes/NA	CHD, CHD mortality, and total mortality
Boekholdt et al. [5]	13,141	58 (45–79)	10.6 y	>4/<0.4	Yes/yes	Yes/yes	CHD, CHD mortality, and total mortality
Ittermann et al. [44]	3883	48.9 (20–79)	8.5 y	NA/<0.25	NA/yes	NA/yes	CHD, CHD mortality, AF, and total mortality
Jongh et al. [21]	1197	75.5 (>65)	10.7 y	>4.5/<0.3	Yes/yes	Yes/yes	CHD, CHD mortality, total, and cardiovascular mortality
Schultz et al. [35]	605	67.8 (50–91)	5 y	>4/<0.4	Yes/yes	NR/NR	Total mortality
Nanchen et al. [33]	5316	75 (70–82)	3.2 y	≥4.5/<0.45	Yes/yes	Yes/yes	CHD, HF, AF, total, and cardiovascular mortality
Asvold et al. [34]	26,475	53.7	12.3 y	>3.6/<0.49	Yes/yes	NR/NR	CHD, CHD mortality, and HF
Ceresini et al. [32]	931	75.5 (>65)	6 y	>4.68/<0.49	Yes/yes	Yes/yes	Total mortality
Hyland et al. [31]	4863	73.5 (>65)	10 y	>4.5/NA	Yes/NA	NR/NA	CHD, cardiovascular mortality, and HF

Shypo: subclinical hypothyroidism; Shyper: subclinical hyperthyroidism; CHD: coronary heart disease; AF: atrial fibrillation; HF: heart failure; TSH: thyroid-stimulating hormone; NR: not reported; NA: not applicable (because the outcome was not examined in the study).

**Table 2:** Stratified and sensitivity analysis of the subclinical hypothyroidism.

Subclinical hypothyroidism and CHD	Summary relative risk (95% CI)^+^	Studies, *n*

*Eligible study model*		
Random effects	1.17 (0.91–1.52)	10
Fixed effects	1.14 (1.04–1.25)	10
*Stratified by mean age*, y		
<65	1.54 (1.00–2.39)	4
≥65	0.96 (0.84–1.10)	6
<60	1.54 (1.00–2.39)	4
60–79.9	0.96 (0.84–1.09)	5
≥80	1.18 (0.59–2.36)	1
*Stratified by TSH*, mIU/L		
4.5–9.9	1.07 (0.67–1.70)	4
10–19.9	1.38 (0.68–2.78)	4
4.5-6.9	0.99 (0.84–1.17)	2
7.0–9.9	0.92 (0.66–1.27)	2
*Adjustments*		
Adjusted analyses or matching	1.17 (0.91–1.52)	10
Adjusted for cardiovascular risk factors	1.09 (0.71–1.65)	6
*Excluding studies*		
Exclusion of studies with thyroid hormone recipients	1.02 (0.92–1.14)	7
Exclusion of studies with particular population [42]	1.17 (0.89–1.54)	9

Subclinical hypothyroidism and total mortality	Summary relative risk (95% CI)^+^	Studies, *n*

*Eligible study model*		
Random effects	1.02 (0.93–1.13)	11
Fixed effects	1.03 (0.95–1.11)	11
*Stratified by mean age*, y		
<65	1.16 (0.96–1.40)	2
≥65	0.98 (0.87–1.10)	9
<60	1.16 (0.96–1.40)	2
60–79.9	1.02 (0.94–1.11)	8
≥80	0.52 (0.25–1.08)	1
*Stratified by TSH*, mIU/L		
4.5–9.9	1.05 (0.80–1.36)	2
10–19.9	1.25 (0.67–2.36)	2
4.5-6.9	0.92 (0.62–1.35)	1
7.0–9.9	1.06 (0.55–2.04)	1
*Adjustments*		
Adjusted analyses or matching	1.02 (0.93–1.13)	11
Adjusted for cardiovascular risk factors	1.06 (0.97–1.15)	5
*Excluding studies*		
Exclusion of studies with thyroid hormone recipients	1.01 (0.90–1.15)	8
Exclusion of studies with particular population [42]	1.04 (0.97–1.13)	10

Subclinical hypothyroidism and cardiovascular mortality	Summary relative risk (95% CI)^+^	Studies, *n*

*Eligible study model*		
Random effects	1.06 (0.77–1.45)	8
Fixed effects	1.13 (0.98–1.30)	8
*Stratified by mean age*, y		
<65	2.14 (1.43–3.22)	1
≥65	1.00 (0.79–1.26)	7
<60	2.14 (1.43–3.22)	1
60–79.9	1.08 (0.90–1.29)	6
≥80	0.42 (0.11–1.63)	1
*Stratified by TSH*, mIU/L		
4.5–9.9	1.03 (0.73–1.45)	3
10–19.9	1.25 (0.81–1.95)	3
4.5-6.9	0.83 (0.31–2.21)	2
7.0–9.9	1.06 (0.73–1.54)	2
*Adjustments*		
Adjusted analyses or matching	1.06 (0.77–1.45)	8
Adjusted for cardiovascular risk factors	1.02(0.52–2.00)	4
*Excluding studies*		
Exclusion of studies with thyroid hormone recipients	0.86 (0.56–1.32)	4
Exclusion of studies with particular population [42]	1.11 (0.81–1.52)	7

CHD: coronary heart disease; TSH: thyroid-stimulating hormone. ^+^Relative risk from meta-analysis using random-effects model.

**Table 3:** Stratified and sensitivity analysis of the subclinical hyperthyroidism.

Subclinical hyperthyroidism and CHD	Summary relative risk (95% CI)^+^	Studies, *n*

*Eligible study model*		
Random effects	1.20 (1.02–1.42)	9
Fixed effects	1.20 (1.02–1.41)	9
*Stratified by mean age*, y		
<65	1.32 (0.98–1.77)	4
≥65	1.14 (0.87–1.50)	5
<60	1.32 (0.98–1.77)	4
60–79.9	1.14 (0.85–1.51)	4
≥80	1.19 (0.49–2.94)	1
*Stratified by TSH*, mIU/L		
0.1–0.44	0.62 (0.21–1.86)	1
<0.1	0.96 (0.33–2.80)	1
*Adjustments*		
Adjusted analyses or matching	1.20 (1.02–1.42)	9
Adjusted for cardiovascular risk factors	1.18 (0.94–1.49)	6
*Excluding studies*		
Exclusion of studies with antithyroid drug recipients	1.18 (0.98–1.41)	7
Exclusion of studies with particular population [42]	1.21 (1.02–1.45)	8

Subclinical hyperthyroidism and total mortality	Summary relative risk (95% CI)^+^	Studies, *n*

*Eligible study model*		
Random effects	1.27 (1.07–1.51)	10
Fixed effects	1.36 (1.22–1.51)	10
*Stratified by mean age*, y		
<65	1.62 (1.37–1.90)	2
≥65	1.17 (0.98–1.40)	8
<60	1.62 (1.37–1.90)	2
60–79.9	1.18 (0.96–1.44)	7
≥80	1.08 (0.60–1.93)	1
*Stratified by TSH*, mIU/L		
0.1–0.44	0.45 (0.12–1.76)	1
<0.1	1.74 (0.78–3.87)	1
*Adjustments*		
Adjusted analyses or matching	1.27 (1.07–1.51)	10
Adjusted for cardiovascular risk factors	1.23 (0.94–1.63)	5
*Excluding studies*		
Exclusion of studies with antithyroid drug recipients	1.26 (1.04–1.53)	8
Exclusion of studies with particular population [42]	1.29 (1.07–1.54)	9

Subclinical hyperthyroidism and cardiovascular mortality	Summary relative risk (95% CI)^+^	Studies, *n*

*Eligible study model*		
Random effects	1.12 (0.84–1.50)	7
Fixed effects	1.10 (0.82–1.47)	7
*Stratified by mean age*, y		
<65	0.84 (0.28–2.50)	1
≥65	1.15 (0.85–1.55)	6
<60	0.84 (0.28–2.50)	1
60–79.9	1.15 (0.84–1.58)	5
≥80	1.11 (0.39–3.17)	1
*Stratified by TSH*, mIU/L		
0.1–0.44	0.47 (0.07–3.27)	1
<0.1	2.16 (0.74–6.34)	1
*Adjustments*		
Adjusted analyses or matching	1.12 (0.84–1.50)	7
Adjusted for cardiovascular risk factors	0.98 (0.65–1.47)	4
*Excluding studies*		
Exclusion of studies with antithyroid drug recipients	1.13 (0.81–1.58)	5
Exclusion of studies with particular population [42]	1.12 (0.83–1.52)	6

CHD: coronary heart disease; TSH: thyroid-stimulating hormone. ^+^Relative risk from meta-analysis using random-effects model.

**Table 4:** Stratified and sensitivity analysis of the subclinical thyroid dysfunction.

Subclinical hypothyroidism and CHD mortality	Summary relative risk (95% CI)^+^	Studies, *n*

*Eligible study model*		
Random effects	1.37 (1.03–1.84)	6
Fixed effects	1.47 (1.22–1.77)	6
*Stratified by mean age*, y		
<65	1.54 (1.11–2.15)	3
≥65	0.98 (0.60–1.62)	3
<60	1.54 (1.11–2.15)	3
60–79.9	1.00 (0.57–1.74)	2
≥80	0.93 (0.30–2.86)	1
*Adjustments*		
Adjusted analyses or matching	1.37 (1.03–1.84)	6
Adjusted for cardiovascular risk factors	1.18 (0.83–1.67)	2
*Excluding studies*		
Exclusion of studies with thyroid hormone recipients	1.17 (0.89–1.53)	5
Exclusion of studies with particular population [42]	1.40 (1.03–1.91)	5

Subclinical hyperthyroidism and CHD mortality	Summary relative risk (95% CI)^+^	Studies, *n*

*Eligible study model*		
Random effects	1.45 (1.12–1.86)	6
Fixed effects	1.42 (1.11–1.83)	6
*Stratified by mean age*, y		
<65	1.49 (1.12–1.98)	3
≥65	1.30 (0.75–2.25)	3
<60	1.49 (1.12–1.98)	3
60–79.9	1.40 (0.79–2.49)	2
≥80	0.55 (0.08–3.77)	1
*Adjustments*		
Adjusted analyses or matching	1.45 (1.12–1.86)	6
Adjusted for cardiovascular risk factors	1.52 (1.06–2.18)	3
*Excluding studies*		
Exclusion of studies with thyroid hormone recipients	1.41 (1.05–1.89)	5
Exclusion of studies with particular population [42]	1.47 (1.14–1.90)	5

^+^Relative risk from meta-analysis using random-effects model.
