# Supplementary material for: Relationship between Subclinical Thyroid Dysfunction and the Risk of Cardiovascular Outcomes: A Systematic Review and Meta-Analysis of Prospective Cohort Studies
Source: Int J Endocrinol. 2017 Aug 31;2017:8130796. doi: 10.1155/2017/8130796 (PMC5610794; doi:10.1155/2017/8130796)
Supplement: Supplementary file 1 — Supplemental Material Table 1. Quality Assessment of Included Studies. Supplemental Material Table 2. Newcastle Ottawa Quality Assessment Scale for Cohorts of included studies. Supplemental Material Table 3.SUMMARY OF FINDINGS FOR THE MAIN COMPARISON. Supplemental Material Table 4: SUMMARY OF FINDINGS FOR THE MAIN COMPARISON. [file 8130796.f1.docx]

| Supplemental Material Table 1. Quality Assessment of Included Studies | | | | | | | |
| --- | --- | --- | --- | --- | --- | --- | --- |
| First Author, Year | Population studied* | Formal Adjudication Procedures for CHD+ | Formal Adjudication Procedures for Cardiovascular Mortality+ | Formal Adjudication Procedures for HF+ | Formal Adjudication Procedures for AF+ | Adjudication without Knowledge of Thyroid Status | Adjustments |
| Parle et al., 2001 ^43^ | Population-based | No | No | NA | NA | Yes | Age and sex |
| Gussekloo et al., 2004 ^42^ | Population-based | No | Yes | NA | NA | Yes | Sex and education |
| Rodondi et al., 2005 ^41^ | Population-based | Yes | Yes | Yes | NA | Yes | Age, sex, race, smoking, diabetes mellitus, prevalent cardiovascular disease , use of thyroid hormone, BP, TC |
| Walsh et al., 2005 ^24^ | Population-based | No | No | NA | NA | No | Age, sex, BMI, smoking, diabetes, TC, BP, exercise, and thyroid disease |
| Cappola et al., 2006 ^23^ | Population-based | Yes | Yes | NA | No | Yes | Age, sex, race, smoking, diabetes, thyroid medication, LDL, hypertension, BMI and CRP |
| Bauer et al., 2007 ^40^ | Population-based | NA | Yes | NA | NA | Yes | Age, weight, thyroid hormone or estrogen use，history of hyperthyroidism |
| Rodondi et al., 2008 ^39^ | Population-based | NA | NA | Yes | NA | Yes | Age, gender, race, smoking, diabetes mellitus, hypertension, BMI, LDL, HDL. |
| Razvi et al., 2010 ^38^ | Population-based | Yes | NA | NA | NA | NR | Social class, body weight, history of cerebrovascular disease, diabetes , smoking and BP |
| Boekholdt et al., 2010 ^5^ | Population-based | Yes | NA | NA | NA | NR | Gender, age, smoking, diabetes , BP, LDL and HDL |
| Ittermann et al., 2010 ^44^ | Population-based | No | NA | NA | No | NR | Age, sex, BMI, smoking, MI,  Hypertension, diabetes,  Cholesterol and fibrinogen |
| Jongh et al., 2011 ^21^ | Population-based | No | No | NA | NA | NR | Age, gender, alcohol, smoking , physical activity, BMI, mean arterial pressure, heart rate and TC |
| Schultz et al., 2011 ^35^ | Population-based | No | No | NA | NA | No | Sex, age, hypertension, diabetes and smoking |
| Nanchen et al., 2012 ^33^ | Population-based | No | No | Yes | Yes | NR | Age, sex, education, history of CVD, diabetes, BMI, smoking, BP, LDL, creatinine, and blocker and antiarrhythmic use. |
| Asvold et al., 2012 ^34^ | Population-based | No | NA | No | NA | No | Age, gender, smoking and BMI |
| Ceresini et al., 2013 ^32^ | Population-based | NA | NA | NA | NA | NR | Age, sex, and BMI |
| Hyland et al., 2013 ^31^ | Population-based | Yes | NA | Yes | NA | Yes | Age, sex and race |

CHD =coronary heart disease; AF= Atrial Fibrillation; HF= Heart Failure; BMI=body mass index; TC=total cholesterol; BP=blood pressure; HDL= high-density lipoprotein;

LDL= low-density lipoprotein; CRP=C-reactive protein; NR= not reported; NA= not applicable (because the outcome was not examined in the study).

+ A formal adjudication procedure was defined as having clear outcome criteria that were reviewed by experts for each potential case.

* A population-based study was defined as a random sample of the general population.

**Supplemental Material Table 2. Newcastle Ottawa Quality Assessment Scale for Cohorts of included studies**

| Study | Selection | Comparability | Outcome | Total |
| --- | --- | --- | --- | --- |
| Parle et al., 2001 ^43^ | *** | ** | *** | 8/9 |
| Gussekloo et al., 2004 ^42^ | *** | ** | ** | 7/9 |
| Rodondi et al., 2005 ^41^ | **** | ** | ** | 8/9 |
| Walsh et al., 2005 ^24^ | **** | ** | ** | 8/9 |
| Cappola et al., 2006 ^23^ | **** | ** | ** | 8/9 |
| Bauer et al., 2007 ^40^ | **** | * | *** | 8/9 |
| Rodondi et al., 2008 ^39^ | *** | ** | *** | 8/9 |
| Razvi et al., 2010 ^38^ | *** | ** | ** | 7/9 |
| Boekholdt et al., 2010 ^5^ | **** | ** | * | 7/9 |
| Ittermann et al., 2010 ^44^ | *** | ** | ** | 7/9 |
| Jongh et al., 2011 ^21^ | *** | ** | ** | 7/9 |
| Schultz et al., 2011 ^35^ | *** | ** | ** | 7/9 |
| Nanchen et al., 2012 ^33^ | *** | ** | ** | 7/9 |
| Asvold et al., 2012 ^34^ | *** | ** | ** | 7/9 |
| Ceresini et al., 2013 ^32^ | *** | ** | ** | 7/9 |
| Hyland et al., 2013 ^31^ | *** | ** | ** | 7/9 |

| **Supplemental Material Table 3.SUMMARY OF FINDINGS FOR THE MAIN COMPARISON** | | | | | | |
| --- | --- | --- | --- | --- | --- | --- |
| Subclinical hypothyroidism compared to euthyroidism for cardiovascular outcomes | | | | | | |
| Patient or population: patients with cardiovascular outcomes Intervention: subclinical hypothyroidism Comparison: euthyroidism | | | | | | |
| Outcomes | Illustrative comparative risks* (95% CI) | | Relative effect (95% CI) | No of Participants (studies) | Quality of the evidence (GRADE) |  |
|  | Assumed risk | Corresponding risk |  |  |  |  |
|  | euthyroidism | Subclinical hypothyroidism |  |  |  |  |
| CHD | 85 per 1000 | 100 per 1000 (78 to 130) | RR 1.17  (0.91 to 1.52) | 57396 (10 studies) | ⊕⊕⊝⊝ low^1,2^ |  |
| Total Mortality | 184 per 1000 | 188 per 1000 (171 to 208) | RR 1.02  (0.93 to 1.13) | 29191 (11 studies) | ⊕⊕⊕⊝ moderate^2^ |  |
| Cardiovascular Mortality | 96 per 1000 | 102 per 1000 (74 to 139) | RR 1.06  (0.77 to 1.45) | 17893 (8 studies) | ⊕⊕⊝⊝ low^1,2^ |  |
| CHD Mortality | 31 per 1000 | 42 per 1000 (31 to 56) | RR 1.37  (1.03 to 1.84) | 43661 (6 studies) | ⊕⊕⊕⊝ moderate^2^ |  |
| Heart Failure | 42 per 1000 | 49 per 1000 (37 to 66) | RR 1.17  (0.87 to 1.57) | 38541 (4 studies) | ⊕⊕⊝⊝ low^1,2^ |  |
| Atrial Fibrillation | 156 per 1000 | 164 per 1000 (142 to 189) | RR 1.05  (0.91 to 1.21) | 8219 (2 studies) | ⊕⊕⊝⊝ low^2,3^ |  |
| *The basis for the assumed risk (e.g. the median control group risk across studies) is provided in footnotes. The corresponding risk (and its 95% confidence interval) is based on the assumed risk in the comparison group and the relative effect of the intervention (and its 95% CI). CI: Confidence interval; RR: Risk ratio; | | | | | | |
| GRADE Working Group grades of evidence High quality: Further research is very unlikely to change our confidence in the estimate of effect.  Moderate quality: Further research is likely to have an important impact on our confidence in the estimate of effect and may change the estimate. Low quality: Further research is very likely to have an important impact on our confidence in the estimate of effect and is likely to change the estimate. Very low quality: We are very uncertain about the estimate. | | | | | | |
| ^1^ Heterogeneity is big ^2^ Wide CI crossing the line of no effect (-1)  ^3^ high risk of outcome reporting bias | | | | | | |

| **Supplemental Material Table 4: SUMMARY OF FINDINGS FOR THE MAIN COMPARISON** | | | | | | |
| --- | --- | --- | --- | --- | --- | --- |
| Subclinical hyperthyroidism compared to euthyroidism for cardiovascular outcomes | | | | | | |
| Patient or population: patients with cardiovascular outcomes Intervention: subclinical hyperthyroidism Comparison: euthyroidism | | | | | | |
| Outcomes | Illustrative comparative risks* (95% CI) | | Relative effect (95% CI) | No of Participants (studies) | Quality of the evidence (GRADE) |  |
|  | Assumed risk | Corresponding risk |  |  |  |  |
|  | euthyroidism | Subclinical hyperthyroidism |  |  |  |  |
| CHD | 78 per 1000 | 93 per 1000 (79 to 111) | RR 1.20  (1.02 to 1.42) | 53214 (9 studies) | ⊕⊕⊕⊝ moderate^2^ |  |
| Total Mortality | 174 per 1000 | 221 per 1000 (186 to 263) | RR 1.27  (1.07 to 1.51) | 26860 (10 studies) | ⊕⊕⊕⊝ moderate^1^ |  |
| Cardiovascular Mortality | 105 per 1000 | 118 per 1000 (88 to 157) | RR 1.12  (0.84 to 1.50) | 12773 (7 studies) | ⊕⊕⊕⊝ moderate^2^ |  |
| CHD Mortality | 29 per 1000 | 42 per 1000 (32 to 53) | RR 1.45 (1.12 to 1.86) | 43622 (6 studies) | ⊕⊕⊕⊝ moderate^2^ |  |
| Heart Failure | 37 per 1000 | 57 per 1000 (32 to 100) | RR 1.54  (0.87 to 2.71) | 32410 (3 studies) | ⊕⊕⊝⊝ low^1,2^ |  |
| Atrial Fibrillation | 123 per 1000 | 175 per 1000 (85 to 360) | RR 1.42  (0.69 to 2.92) | 10553 (3 studies) | ⊕⊕⊝⊝ low^1,2^ |  |
| *The basis for the assumed risk (e.g. the median control group risk across studies) is provided in footnotes. The corresponding risk (and its 95% confidence interval) is based on the assumed risk in the comparison group and the relative effect of the intervention (and its 95% CI). CI: Confidence interval; RR: Risk ratio; | | | | | | |
| GRADE Working Group grades of evidence High quality: Further research is very unlikely to change our confidence in the estimate of effect.  Moderate quality: Further research is likely to have an important impact on our confidence in the estimate of effect and may change the estimate. Low quality: Further research is very likely to have an important impact on our confidence in the estimate of effect and is likely to change the estimate. Very low quality: We are very uncertain about the estimate. | | | | | | |
| ^1^ Heterogeneity is big ^2^ Wide CI crossing the line of no effect (-1) | | | | | | |
